# Phosphate Limitation Increases Content of Protease Inhibitors in the Cyanobacterium *Microcystis aeruginosa*

**DOI:** 10.3390/toxins12010033

**Published:** 2020-01-06

**Authors:** Christian Burberg, Thomas Petzoldt, Eric von Elert

**Affiliations:** 1 Workgroup Aquatic Chemical Ecology, Institute for Zoology, University of Cologne, 50674 Cologne, Germany; evelert@uni-koeln.de; 2 Institute of Hydrobiology, Technische Universität (TU) Dresden, 01062 Dresden, Germany; thomas.petzoldt@tu-dresden.de

**Keywords:** eutrophication, freshwater, protease inhibitors, toxin production, cyanotoxins

## Abstract

Increased anthropogenic nutrient input has led to eutrophication of lakes and ponds, resulting worldwide in more frequent and severe cyanobacterial blooms. In particular, enhanced availability of phosphorus (P) can promote cyanobacterial mass developments and may affect the content of secondary metabolites in cyanobacteria, such as protease inhibitors (PIs). PIs are common among cyanobacteria and have been shown to negatively affect herbivorous zooplankton. Here, we test the hypothesis that P-limitation reduces the growth of *Microcystis,* but increases the content of PIs. In batch culture experiments with eight different initial phosphate concentrations (5–75 µM) we determined growth, stoichiometry, and PI content of *Microcystis aeruginosa* NIVA Cya 43. This strain produces the protease inhibitor BN920 that is converted by chlorination to CP954, which constitutes the major PI in this strain. C:N:P-ratios of the biomass indicated variation of P-limitation with treatment and time. When normalized to biomass, the PI content varied up to nearly nineteen-fold with treatment and time and was highest in the low-P treatments, especially during the mid-exponential growth phase. However, these effects were alleviated under nitrogen co-limitation. The content of CP954 showed an inverse u-shaped response to growth rate and C:N-ratio of the cyanobacterial biomass, whereas it increased with cyanobacterial C:P. The results indicate that P-limitation supports a higher content of defensive PIs and may indirectly foster cyanobacterial blooms by increasing the negative interference of cyanobacteria with their consumers.

## 1. Introduction

The frequency of cyanobacterial blooms in lakes and ponds has increased due to eutrophication and rising water temperatures [1,2]. Many bloom-forming cyanobacteria, such as *Microcystis*, produce a wide range of toxic secondary metabolites that pose an eminent threat to the public health [3,4] and the environment by impacting the food web and ecosystem functioning [5,6,7]. This results in substantial economic costs as cyanobacterial blooms reduce lake services like drinking water quality, recreational usage, or fisheries [8].

Nutrient loading has been considered a major cause for the increasing number of cyanobacterial blooms. In freshwater systems, phosphorus (P) is traditionally seen as the limiting factor for productivity [9], whereas nitrogen (N) is regarded as the limiting factor in marine ecosystems [10]. Phosphate (PO_4_^3−^) is the most common P form on earth [11], but it is often immobilized and has therefore a low bioavailability [12]. Through the extensive use of P-containing washing agents and P-containing fertilizers in agriculture, P is washed into ponds and lakes, where it promotes the development of cyanobacterial blooms. A prominent example is Lake Erie, where P-input through the Maumee River has repeatedly led to blooms of *Microcystis* in the western basin of the lake [13]. Reduction of P is therefore a widely used approach to reduce eutrophication and thus lower the probability of cyanobacterial blooms [9,14]. Despite attempts to reduce the input of P into freshwater systems, the effects of high P-loads are still visible in many aquatic systems, and climate change may even increase the P input in the future [13,15]. However, recent research demonstrated that P is not always the only factor that determines the formation of cyanobacterial blooms but that N and the N:P-ratio are also important, and, at least in some cases, a reduction of N and P seems necessary to suppress cyanobacterial bloom formation [16,17,18].

Increasing nutrient concentrations do not only promote the development of blooms, they may also affect the toxicity of such blooms. A number of studies have investigated the effects of environmental factors such as pH, light, iron, and major nutrients like N and P on the production and content of secondary metabolites [19,20,21,22,23,24]. Most of these studies focused on microcystin (MCs) and MC-producing cyanobacteria. MCs are the most prominent group of cyanobacterial metabolites and are highly toxic, even for humans [3]. However, besides MCs, there are a variety of other cyanobacterial secondary metabolites, including the huge group of protease inhibitors (PIs) [25].

PIs are widespread among cyanobacterial taxa and were isolated from many cyanobacterial blooms [26,27]. In drinking water treatment plants, PIs can reach loadings that are comparable to that of MCs, and these loadings are strongly correlated with the abundance of cyanobacteria [28]. This underlines the importance to analyze these compounds. Cyanopeptolines are a group of potent PIs, of which for example cyanopeptolin 1020 has been shown to inhibit human kallikrein [29]. Ahp-cyclodepsipeptides, including cyanopeptolines, have been reported in field samples [28,30,31,32,33], and more than 200 different Ahp-cyclodepsipeptides have been identified in cyanobacterial strains covering several genera [25], which underlines that they are widespread.

However, PIs might not only pose a threat to human health, but as well affect trophic interactions in freshwater food chains. In lakes and ponds the microcrustacean *Daphnia* is a major consumer of planktonic primary production and serves as an important prey for planktivorous fish. Hence, the trophic transfer efficiency from primary producers to *Daphnia* is key to the trophic transfer of mass and energy in pelagic food webs in freshwaters [34]. Cyanobacterial PIs reduce this trophic transfer by specifically inhibiting digestive proteases in the gut of *Daphnia* and thereby decrease growth of this herbivore [35,36,37]. This is in accordance with findings of von Elert et al. [38], who demonstrated that chymotrypsin and trypsin account for 80% of the total digestive protease activity in the gut of *Daphnia*.

More detailed investigations of the interaction of cyanobacterial PIs with *Daphnia* have been performed with a strain of *Microcystis aeruginosa* that produces two cyanopeptolins that specifically target chymotrypsins in the gut of *Daphnia*; these inhibitors have been identified as cyanopeptolin 954 (CP954) and nostopeptin 920 (BN920) [39]. Although many aspects of the interference of these two cyanopeptolin PIs with *Daphnia* physiology are understood, effects of nutrient concentrations on the cellular content of these two inhibitors have not been investigated yet (except for [24]), although this strain is ideally suited for such approaches, as it does not produce microcystins [39].

Changes in resource availability may affect cyanobacterial blooms directly by providing more nutrients, but also indirectly by changing their cellular toxin content and thus altering the negative interference of cyanobacteria with their grazers [21,24]. In this study, we evaluated the effect of P-availability on the growth and the content of the two cyanopeptolin PIs CP954 and BN920 in *M. aeruginosa*. In batch culture growth experiments with eight different initial P-concentrations, we quantified cell numbers and particulate organic carbon over time and used a logistic growth model to determine growth rates at all time points. We further determined C:N:P stoichiometry of the cyanobacterial biomass and quantified the content of CP954 and BN920 using UPLC coupled to a high-resolution mass spectrometer. The two PIs CP954 and BN920 are N-rich secondary metabolites that do not contain P. We hypothesized that P-limitation of the cyanobacterium would result in higher availability of N for synthesis and hence of higher biomass content of CP954 and BN920.

## 2. Results

In batch culture experiments, different initial phosphate (PO_4_^3−^) concentrations were tested for their effects on the growth performance, stoichiometry and content of two N-rich protease inhibitors, nostopeptin 920 (BN920) and cyanopeptolin 954 (CP954), in *Microcystis aeruginosa* strain NIVA Cya 43.

### 2.1. Growth Performance of M. aeruginosa under Different PO_4_^3−^ Conditions

**Figure 1:**
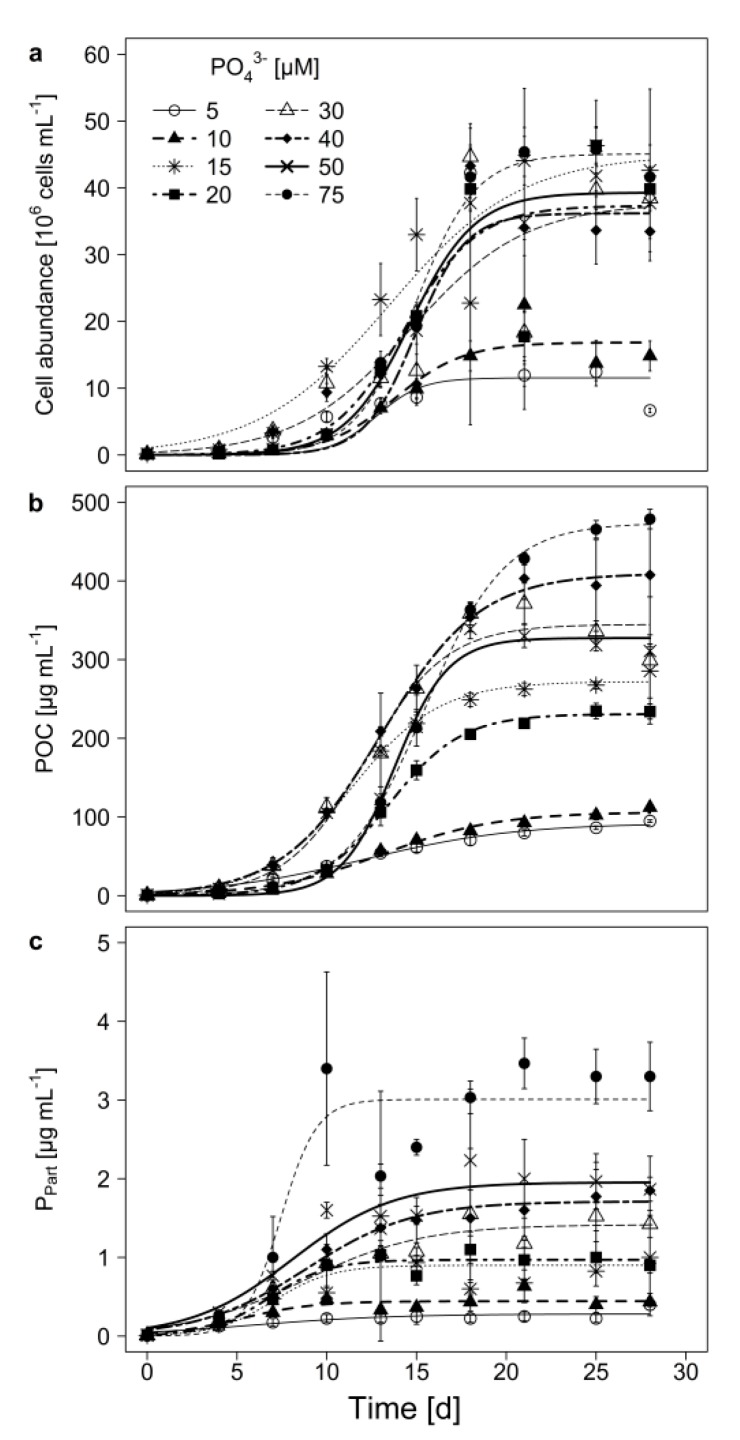
(**a**) Cell abundance, (**b**) particulate organic carbon (POC), and (**c**) particulate phosphorus (P_part_), in batch culture growth experiments of *M. aeruginosa* with 8 different initial PO_4_^3−^-concentrations. Mean ± SD, n = 3–4. Displayed curves were fitted using a logistic growth model. Model parameters for (**a**) and (**b**) are given in Table 1; Table 2.

Under all tested PO_4_^3−^-concentrations, *M. aeruginosa* showed logistic growth, regardless whether growth was measured as cell abundance or particular organic carbon (POC; Figure 1a,b). The carrying capacity (K; Table 1 and Table 2) was lowest at 5 µM P and reached only 20% of the biomass that was obtained with the highest P-concentration. This indicates a growth limitation at very low P-concentrations. The strength of the inhibition seems to decrease with increasing P-concentration, as both K and maximal growth rate (µ_max_) increased with initial P-concentration (Table 1; Table 2). Except for the 30 µM P treatment, biomass determined as POC, remained stable towards the end of the experiment.

In all treatments, the particulate phosphorus (P_part_) increased during the experiment (Figure 1c). The linear mixed model shows that especially the sampling day as well as the treatment x day interaction had an impact on P_part_, while the treatment alone had no significant effect on P_part_ (Table 3). However, at the end of the experiment (on day 28), P_part_ was highest at the highest P-concentration and lowest at 5 µM P (Figure 1c, Tukey HSD after one-way ANOVA, F = 28.78, *p* < 0.001).

### 2.2. Stoichiometry of M. aeruginosa Under Different PO_4_^3−^ Conditions

**Figure 2:**
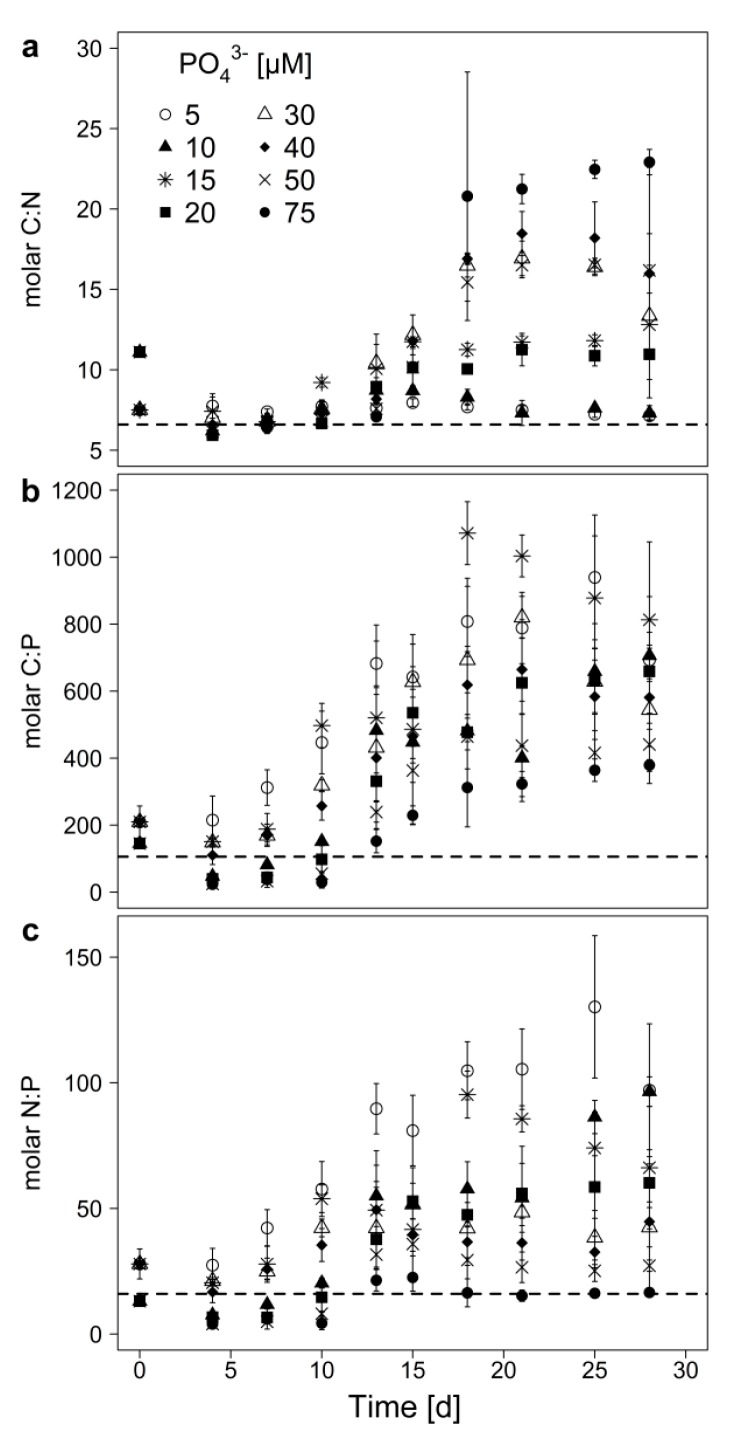
Elemental molar ratios (**a**) C:N, (**b**) C:P, and (**c**) N:P of the biomass of *M. aeruginosa* grown on 8 different initial of PO_4_^3−^-concentrations. Mean ± SD, n = 3–4. The dashed horizontal lines indicate the respective Redfield ratio.

The elemental molar ratios (C:N, C:P and N:P) of the cyanobacterial biomass showed high flexibility throughout the experiment and were affected by the treatment (P-concentration), the sampling day, and the interaction of treatment and sampling day (Table 3). The C:P-ratio increased independent from the initial P-concentration up to ratios of 300 and more (Figure 2b), indicating an ongoing P-limitation in all treatments. The highest C:P-ratios were reached with 5 and 15 µM initial P, while the lowest were found in the 75 µM treatment. The C:N-ratio increased with initial P-concentrations greater than 15 µM (Figure 2a), while below 15 µM P the C:N-ratio remained around the Redfield ratio (C:N = 6.6). This indicates N-limitation (C:N > 20) at the highest and no N-limitation at the low P-concentrations. The N:P-ratio increased especially if the cyanobacterium was grown at low P-availability, but was around the Redfield ratio at the highest P-concentration (Figure 2c). This points at an excess of N, which might be available for the production of secondary metabolites under low P.

**Figure 3:**
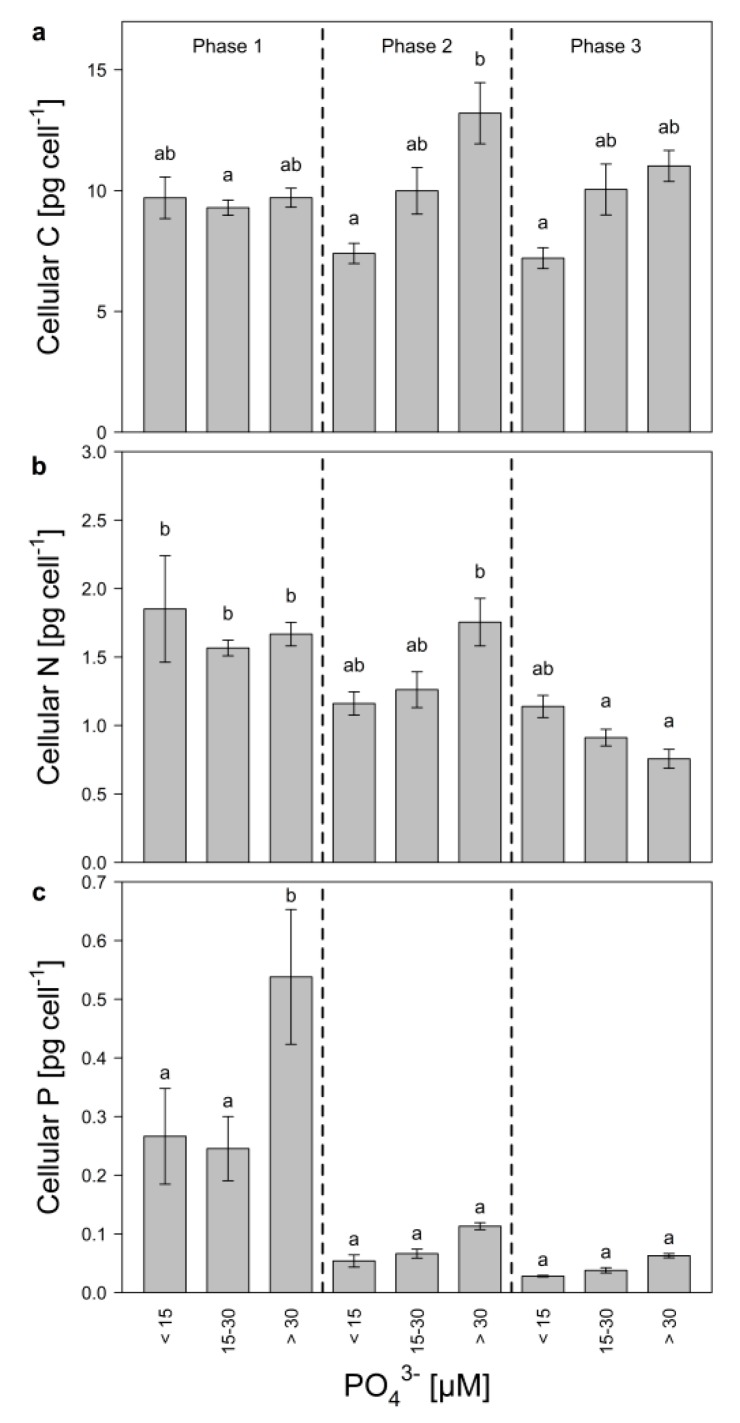
Cellular quota of (**a**) carbon, (**b**) nitrogen, and (**c**) phosphorus of *M. aeruginosa* during early exponential (phase 1), mid-exponential (phase 2), and late growth (phase 3) in treatments with low (<15 µM), medium (15–30 µM) and high (>30 µM) initial P-concentrations. Bars denoted by the same letter are not significantly different (*p* ≥ 0.05), whiskers indicate standard errors.

Cellular carbon (C) was grouped according to growth phase and P-regime (low, intermediate, and high), to estimate if changes in cell size of *Microcystis* occurred (Figure 3a). Even though not significant, cellular C changed slightly, depending on P-regime and growth phase. Especially during the mid-exponential phase, cellular C was significantly higher in the high P-treatments compared to the low P-treatments, indicating that during this growth phase, cell size increased with increasing P-concentrations. Additionally, cellular C decreased from the mid-exponential to the late growth phase in the high P-treatments, which might suggest that *Microcystis* became light-limited. Cellular N decreased significantly in high and medium P-treatments during growth (Figure 3b) and was nearly 1.5-fold higher in the low P-treatments (<15 µM) than in the high P-treatments (>30 µM) during the late growth phase. Cellular P increased with initial P during the early exponential phase (Figure 3c) and decreased later on in all treatments pointing at initial P-storage.

### 2.3. Inhibitor Content of M. aeruginosa Under Different PO_4_^3−^ Conditions

*M. aeruginosa* constitutively produced the two PIs, BN920 and CP954, which accounted for 2.5% to 10% of the total cyanobacterial carbon during the experiment, depending on the day and the treatment. This indicates the high biological relevance of PIs for this *M. aeruginosa* strain.

**Figure 4:**
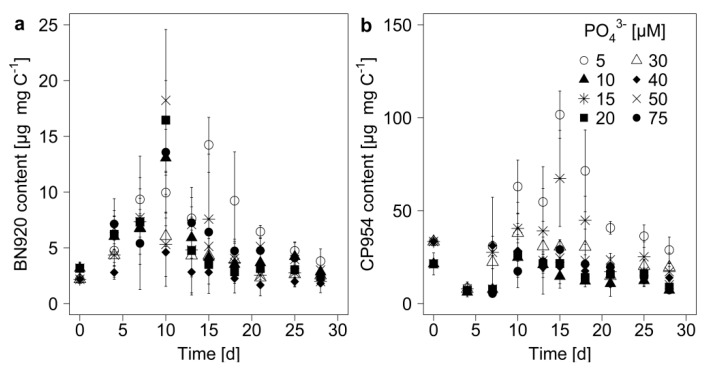
Protease inhibitor content of (**a**) nostopeptin 920 (BN920) and (**b**) cyanopeptolin 954 (CP954) in the biomass of *M. aeruginosa*. The inhibitor content was normalized to the particulate organic carbon (mg C), which served as a proxy for cyanobacterial biomass. Mean ± SD, n = 3–4.

**Figure 5:**
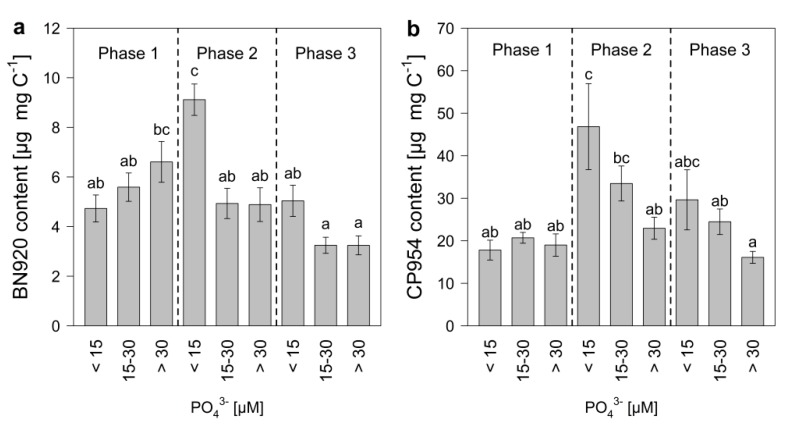
Protease inhibitor content of (**a**) nostopeptin 920 (BN920) and (**b**) cyanopeptolin 954 (CP954) of *M. aeruginosa* during early exponential (phase 1), mid-exponential (phase 2), and late growth (phase 3) in treatments with low (<15 µM), medium (15–30 µM) and high (>30 µM) initial P-concentrations. Bars denoted by the same letter are not significantly different (*p* ≥ 0.05), whiskers indicate standard errors.

The PI content of the cyanobacterium was determined by normalizing the measured PI amount to POC, which served as a measure of cyanobacterial biomass. The PI content of *M. aeruginosa* changed during the experimental time, with the highest contents between day 10 and 18 (Figure 4). The patterns for the single inhibitors were similar, even though changes in the content of BN920 came slightly ahead of changes in the CP954 content. The content of BN920 varied up to eleven-fold and that of CP954 by nearly nineteen-fold with initial P-concentration, time, and their combined effect (Table 4), showing that the dynamics in the PI content over time differed with initial P-concentration. In particular, the content of CP954, the higher concentrated PI, was highest in the low-P treatments on most days, indicating that low P-concentrations can lead to a higher PI content. When these data were grouped according to growth phase and P-regime (Figure 5), the content of both PIs was maximal in the low P-treatments (<15 µM) during mid-exponential growth phase. Additionally, there was a tendency for the PI content to decrease from low to high P-treatments during mid-exponential and late growth, which was not the case during the early exponential phase.

The PI content per cell showed a similar pattern as the content per cyanobacterial biomass (data not shown), ranging from 14.3 to 199.5 pg cell^−1^ for BN920 and from 52 to 725 pg cell^−1^ for CP954.

### 2.4. Inhibitor Content As a Function of Growth Rate and Stoichiometry

**Figure 6:**
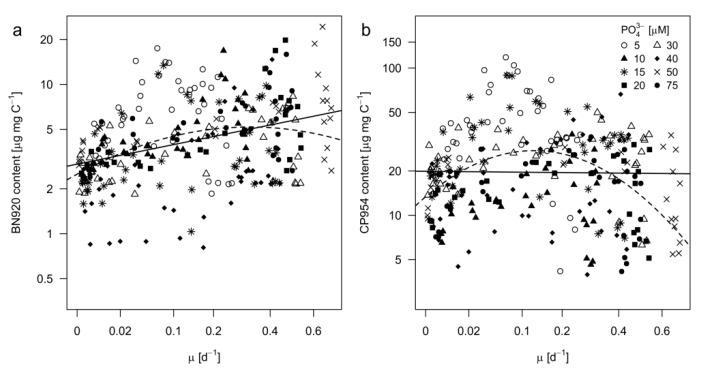
Protease inhibitor content of (**a**) nostopeptin 920 (BN920) and (**b**) cyanopeptolin 954 (CP954) as a function of the growth rate of *M. aeruginosa*. Growth rates were taken from the logistic growth model for POC (see Figure 1b). Each point represents the measured inhibitor content in a single replicate at a single sampling day and the corresponding specific growth rate. Different symbols represent the different treatments. The *x*-axis was square root and the *y*-axis natural logarithmic transformed. Linear (solid lines) and squared (dashed lines) regression models were applied. Equations, R^2^- and *p*-values are shown in Table 5.

It is characteristic for logistic growth that the instantaneous specific growth rate changes over time. In this experiment, specific growth rates were obtained by applying a logistic growth model to the POC data (Figure 1b) and therefore might be referred to as C net production rates. Growth rates were then used to test how the content of each PI behaved as a function of the growth rate of *Microcystis* (Figure 6). For this purpose, mixed effects models with linear and quadratic terms were fitted to the data. In the case of BN920, the quadratic model was slightly better than the linear model in describing the relationship of BN920 and growth rate, as indicated by a smaller AIC (Akaike Information Criterion) and a minimal increase of R^2^ (Table 5). For CP954, the linear model was not significant (*p* = 0.83). Instead, the quadratic model was substantially better suited as indicated by a lower AIC and a higher R^2^. Accordingly, the CP954 content showed a reversed U–shaped (optimum) relationship with the growth rate, reaching a maximum at growth rates of 0.15. However, though significant, the models explained only a low degree of variation within our data (see R^2^, Table 5).

**Figure 7:**
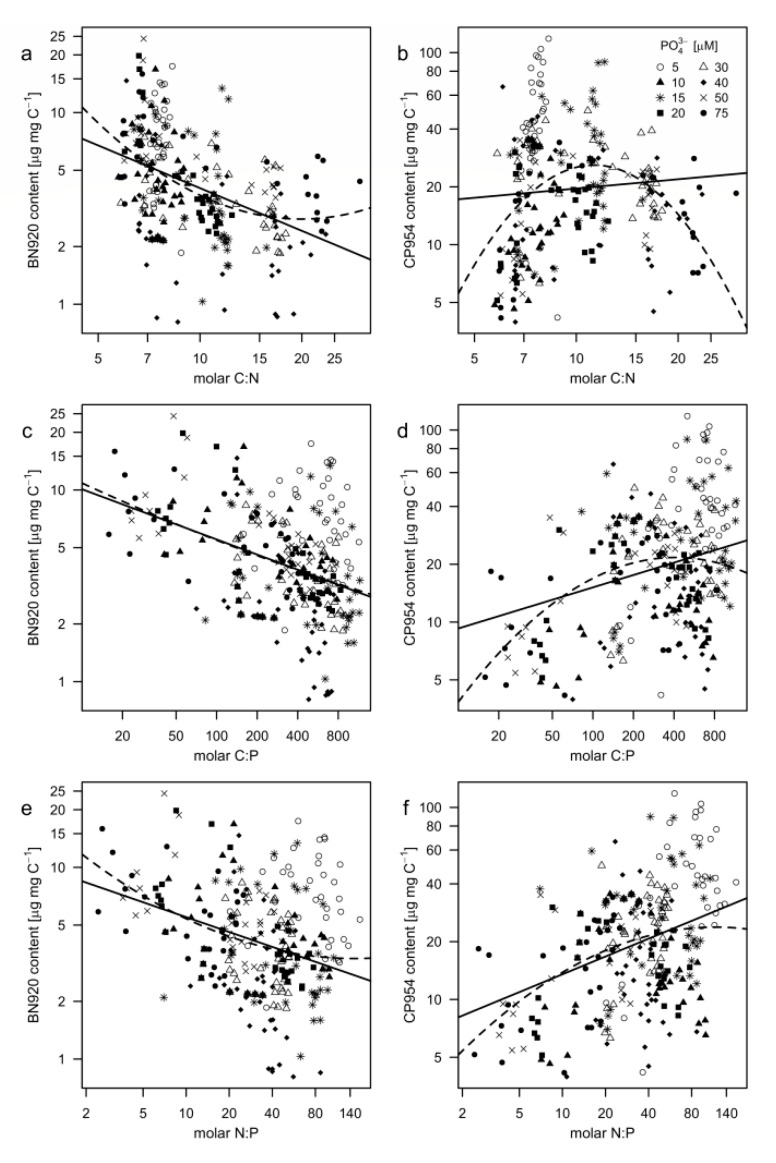
Protease inhibitor content of (**a**,**c**,**e**) nostopeptin 920 (BN920) and (**b**,**d**,**f**) cyanopeptolin 954 (CP954) as a function of the molar ratios between carbon, nitrogen, and phosphorus of *M. aeruginosa*. Each point represents the measured inhibitor content in a single replicate at a single sampling day and the corresponding stoichiometric ratio. Different symbols represent the different treatments. Both axes were natural logarithmic transformed. Linear (solid lines) and squared (dashed lines) regression models were applied. Equations, R^2^-, and *p*-values are shown in Table 5.

The PI content was also plotted as a function of the stoichiometric ratios of the cyanobacterial biomass to test how the stoichiometric ratios affected the PI content of *M. aeruginosa*. Linear models significantly described the relationship between BN920 and the elemental stoichiometric ratios (Table 5), even though the quadratic model was slightly better suited in the case of C:N (lower AIC, higher R^2^). In the case of CP954, all applied models were significant, except for the linear model with C:N. The lower AIC values indicate that the quadratic model described the relationship of CP954 and C:N much better, while the linear model was sufficient to describe the relationship between CP954 and N:P. More specific, the content of BN920 decreased with increasing C:N-ratios (Figure 7a, Table 5); the CP954 content however increased with the C:N-ratio until a C:N-ratio of around 11 and declined at higher C:N-ratios (reversed U-shaped curve; Figure 7b, Table 5). This indicates that N-depletion of the cyanobacterium resulted in decreased PI contents. When plotted against the C:P- (Figure 7c,d; Table 5) and N:P-ratios (Figure 7e,f; Table 5) we found that the contents of BN920 and CP954 were affected differently. While the BN920 content decreased with increasing C:P- and N:P-ratios, the CP954 content increased. As CP954 makes up a larger proportion of the total PI content, a lower P-regime might promote the biomass content of PIs.

## 3. Discussion

The eutrophication of freshwater systems with nitrogen (N) and phosphorus (P) is the main factor promoting cyanobacterial biomass and biovolume [6,27]. In particular, P-loading plays an essential role in lakes and ponds, as many of these systems are limited by P-availability [9]. It has been suggested that global warming will increase bloom frequencies directly by promoting cyanobacterial growth [40] and indirectly via enhanced release of P from sediments [41]. However, it remains controversial to which degree this putatively increased P-availability will shift primary production from P- to N-limitation in freshwater systems. Increased P-availability may be counteracted by regional N deposition from the atmosphere, which has been shown to shift more lakes to P-limitation [42]; on the other hand, internal processes like denitrification may compensate for additional N input [43]. The frequencies of N- or P-limitation in freshwater systems are similar, and quite often co-limitation by N and P is controlling cyanobacterial growth [43,44].

Here we used 5–75 µM initial P-concentrations in batch culture experiments, in which, due to the logistic growth, the initial degree of resource-limitation changes with time. Our results demonstrate that *Microcystis aeruginosa* strain NIVA Cya 43 reached its maximum biomass at the highest P-concentration with biomasses decreasing with decreasing initial P, which indicates a strong P-limitation. This is supported by the very low cellular P contents that were reached in the late growth phase. Except for the lowest P-treatments (5–10 µM), particulate phosphorus (P_part_) had already reached its maximum when biomass was still further increasing. This decoupled increase of biomass points at the well-known rapid uptake of available P and its internal storage as polyphosphate with a subsequent re-mobilization of polyphosphate for further biomass synthesis and growth in cyanobacteria [40,45].

In line with this, we observed increasing C:P-ratios of biomass over time. The inoculum for the batch growth experiment had been pre-cultivated under P-limiting conditions. The initial very low P_part_ content per carbon (0.015 mg P/mg C, which is equivalent to 1.5% per dry weight) matches values reported for P-limited *M. aeruginosa* [45], and the initial C:P values of 145 and 210 (Figure 2b) corroborate that internal polyphosphate pools had been depleted prior to the experiment.

In all treatments, C:P increased over time which corroborated the well-known stoichiometric plasticity of photoautrophs [11]. Initial C:P-ratios roughly matched the Redfield ratio (C:N:P = 106:16:1 [46]), which is an indicator for non-limiting growth conditions. However, over time, C:P-ratios increased and reached maxima ranging from 300 (initially high P) to 1100 (initially low P), which indicates that in all treatments, *M. aeruginosa* was P-limited, though to differing degrees. This is further supported by the cellular P quota, which were lower at low P-concentrations. The only exceptions to this increase over time are C:P-ratios in early samples of high-P treatments, in which C:P-ratios dropped initially. This points at initial luxury consumption of P and a temporary internal P-storage, probably as polyphophate. Cellular N and P quota were also much higher in the early exponential growth phase, underlining that maybe not only P but also N was stored temporary, for example, as cyanophycin [47].

If growth would be limited by P only, other resources should be non-limiting and hence the C:N-ratio should not be deviating from the Redfield ratio (C:N = 6.6). This was the case in the two lowest P-treatments, which indicates that they were only P-limited and that C did not accumulate under severe P-limitation. However, in batch cultures, other factors (e.g., N, light) might become (co-) limiting, especially towards the end of the experiment. The fact that final C:N-ratios increased with increasing initial P might in theory be caused by increased C assimilation. However, as final cellular C quota were not enhanced in high P-treatments, increased final C:N-ratios point at increasing limitation by N. This is supported by the low cellular N content in the high P-treatments. However, in high P-treatments, also the cellular C quota declined, compared to the mid-exponential phase, which points at reduced cell volumes, which suggests light limitation due to self-shading towards the end of the experiment. This indicates that in high P-treatments, in the end, a co-limitation of N and light might have occurred. Between these two extremes of final strong P-limitation (low initial P) and final light- and N-limitation (high initial P), putatively increasing N-co-limitation has occurred with increasing P-concentration, as is suggested by increasing final C:N-ratios.

The production of cyanobacterial secondary metabolites (i.e., toxins and inhibitors) may be linked to the nutrient status of cyanobacteria. If toxins contain the limiting nutrient, cyanobacteria should reduce the production of these metabolites [22] except that these metabolites contribute to nutrient storage (e.g., [24]). Accordingly, the content of microcystins (MCs), N-rich cyanobacterial metabolites, was reduced upon N-limitation [48], and MC production was highest under conditions where N was least limiting [21].

The ecological role of secondary metabolites for cyanobacteria is still not clear [49]. Several functions have been proposed, but many are still lacking real evidence. This is different for the huge group of protease inhibitors (PIs) [25], which tend to be at least as frequent as MCs in natural cyanobacterial blooms [26,27] and which serve as anti-grazer defense against *Daphnia* [36,37,38,50]. Here, the two PIs nostopeptin 920 (BN920) and cyanopeptolin 954 (CP954) were investigated, for which IC_50_ values of 3.1 and 4.5 nM for the inhibition of bovine chymotrypsin have been reported [39], and IC_50_ values for the inhibition of chymotrypsin activity in the gut of *Daphnia* were 5.4 and 7.4 nM respectively [37], which classifies BN920 and CP954 as the most potent inhibitors containing 3-amino-6-hydroxy-2-piperidone (Ahp).

BN920 and CP954 consist of 8 amino acids [39], do not contain P, and have a C:N-ratio of 5.75, so that they can be considered N-rich [22]. Both inhibitors were constitutively present in this strain, which confirms findings for other cyanopeptolins [51]. The fact that a similar ratio of both inhibitors was observed when the cyanobacterium was grown under different degrees of N-limitation [24] suggests a joint biosynthetic pathway with CP954 as the chlorine-containing adduct of BN920.

In our experiment, the PI content was highest in the low-P treatments. Likewise, P-limitation increased the content of N-rich micropeptin PIs in *Microcystis* sp. [52]. Similar effects were reported for the N-rich paralytic shellfish poisoning (PSP) toxin in dinoflagellates [22,53]. This increase in PSP-toxin has been interpreted as evidence for resource-driven toxin synthesis, as P-limitation releases the autotroph from potential N-limitation so that N becomes non-limiting. As a consequence, P-limitation causes a resource-driven increase of N-rich compounds that do not contain P. The same explanation is valid here for a cyanobacterium, where the low P treatments rendered N non-limiting. Such an increase of the content of BN920 and CP954 with increasing N-availability has been reported earlier [24]. With ≥ 20 µM initial P, increasing N-co-limitation was observed towards the end of the experiment, which explains the concomitant decrease in PI content: the carbon nutrient balance hypothesis (CNBH, [54]) predicts that C-rich metabolites are favored under nutrient limitation, whereas in nutrient-replete (here N-replete) environments, more N-based metabolites are produced. Even though, in some cases, the CNBH fails to explain the relationship between secondary metabolite concentrations and nutrient availability (discussed in [55,56]), our results are in accordance with the CNBH.

For MCs, the effects of P-limitation are less clear than those reported here for BN920 and CP954 [22]. This can probably be attributed to the fact that in the latter study, among others, N-fixing cyanobacteria have been considered, in which P-limitation might have reduced N-fixation and thus have caused a decline in MC-content with P-limitation (pers. comm. Van de Waal). For the cyanobacterium *Aphanizomenon*, it was shown that the content of cylindrospermopsin increased with increasing P-concentration, but decreased under P-limitation [57]. However, for *Nostoc* sp. and *Microcystis*, the highest concentration of MC was found under P-reduced conditions [58,59,60].

In our experiments, the PI content showed pronounced temporal variability. In the treatments with ≥ 20 µM initial P, the content of CP954, the major inhibitor, decreased from day 15 onwards. The concurrently increasing N-co-limitation (i.e. increasing C:N-ratios) provides a very plausible explanation for the decrease in PI content after day 15. In treatments with <20 µM initial P, the CP954 content declined as well after day 15, but in a more pronounced way. Here the only explanation is that by day 18 C:P-ratios in these low-P treatments had reached fairly extreme C:P values ranging from 800 to 1000, and that this strong P-limitation might have caused a degradation of CP954 by 50% to 70%. However, we cannot exclude that, depending on culture conditions, release of PIs into the medium has occurred. For MCs, which are known as endotoxins, it is known that they can be found in the surrounding environment due to cell lysis as it occurs during collapse of cyanobacterial blooms [61]. Cell lysis would be associated with the release of cell content into the medium and should thus lead to lower POC values. Such a decline in POC was not observed in our treatments, which suggests that no significant cell lysis has occurred. An exception is the 30 µM P treatment, in which POC declines during the last two samplings. However, this drop in POC is not associated with a decline in inhibitor content at the last two samplings (Figure 3). In conclusion, it is rather improbable that cell lysis has contributed to declines in PI content of *Microcystis* biomass in our experiments.

For P-limitation, effects of growth rate on cyanobacterial secondary metabolites are controversial, sometimes even within the same study. Tonk et al. [49] found that with increasing growth rates, the content of MC and anabaenopeptin increased while the content of cyanopeptolins declined. This was partly corroborated by Long [62], who reported that content and production rate of MC and cyanopeptolins increased with the specific growth rate under P-limitation. As well for P-limitation, but not corroborating these findings, Oh et al. [58] found in chemostat experiments that the MC content of *M. aeruginosa* was highest when the growth rate was lowest. These divergent results indicate that growth rate alone is insufficient to explain the content of secondary metabolites in cyanobacteria, but that it rather depends on which factor controls the growth (e.g., N, P, light). Similar results were also found for the marine dinoflagellate *Alexandrium* [53], suggesting that this dependency on the kind of growth-limitation might not be restricted to cyanobacteria.

In our study, growth rates are based on changes in POC and not on cell numbers. Even though cellular C showed small differences (Figure 3a), POC serves as a good proxy for the cyanobacterial biomass, and therefore growth rates based on POC are well suitable to describe changes in biomass. We found that BN920 significantly increased with growth rate, while for CP954 the content increased with growth rate until 0.15 and declined at higher growth rates (reversed u-shape). This difference is remarkable, as the high chemical identity of both PIs strongly suggests that they share the same biosynthetic pathways with an additional chlorination of BN920, which yields CP954. The fact that the CP954 content exceeds that of BN920 by one order of magnitude strongly suggests that BN920 serves as an intermediate that subsequently undergoes chlorination, probably by a halogenase. The occurrence of chlorinated secondary products is widespread in cyanobacteria [63], and in several cases halogenases have been shown to be involved in their synthesis. Among such cyanobacterial metaoblites are, in addition to CP954, also other chlorinated protease inhibitors [64]. However, both regressions explain only a small degree of data variability (R^2^), which most probably can be attributed to the fact, that the growth rates represent not only treatments with P-limitation but as well, other treatments with N-co-limitation. These two resource-regimes have opposite effects on the content of CP954 and BN920, and it may be hypothesized that they are as well differently related to growth rate, so that no far-reaching conclusions about growth rate effects can be drawn here.

We had hypothesized that P-limitation reduces the growth of *Microcystis,* but increases the content of PIs. Our experiments confirm growth reduction with P-limitation in the *M. aeruginosa* strain NIVA Cya 43 and demonstrate that only under strong P-limitation, a nutrient-driven increase of PI content in cyanobacteria is to be expected, which could lead to enhanced negative effects on grazers like *Daphnia*. Cyanobacteria can be good competitors at low P_i_ due to several alkaline phosphatases and the capability to store P [23]. Still, not all cyanobacteria are good competitors under strong P-limitation [65], and thus cyanobacteria might not be dominating phytoplankton communities under strong P-limitation. More moderate P-limitation would be associated with N-co-limitation that would reduce PI content in cyanobacteria. However, the resulting interference with grazers like *Daphnia* will largely depend on tolerance traits in the grazer community. Standing populations of *Daphnia* harbor pronounced clonal variability with respect to tolerance of cyanobacteria [66] that allows for the reported microevolutionary adaptation of *Daphnia* populations both in time and space [67,68]. Additionally, phenotypic plasticity constitutes another mechanism for acquired tolerance to toxic cyanobacteria. For example, in some cladocerans, transgenerational adaptation has been shown to increase tolerance in zooplankton to toxic dietary cyanobacteria [69,70]. Although the molecular mechanisms driving these maternal effects have received some attention [71,72,73,74], the overall effects of evolution and plasticity of grazers in mitigating resource-driven changes in cyanobacterial toxin content remains to be understood.

## 4. Material and Methods

### 4.1. Culturing Conditions

*Microcystis aeruginosa* NIVA Cya 43 (NORCCA, Norwegian Institute for Water Research, Oslo, Norway) was grown in pre-cultures for 9 days in 1 L Erlenmeyer flasks filled with 400 mL modified WC medium [75]. The culture was kept under constant light (45 ± 3 µmol photons m^−2^ s^−1^) and temperature (20 ± 1 °C) on a horizontal shaker (90 rpm). For the pre-culture we used 10 µM initial phosphate (PO_4_^3−^) to deplete the internal P-reserves of the cyanobacterium. Unlike some other *Microcystis* strains, NIVA Cya 43 does not produce colonies or microcystins, but it does produce cyanopetolin 954 (CP954) and nostopeptin 920 (BN920) [39], which are two nitrogen (N)-rich protease inhibitors (PIs)**.** The cultures were not axenic, and heterotrophic bacteria made up just a small percentage compared to the cyanobacterial biomass [24].

In the experiment, eight initial PO_4_^3−^-concentrations in a range from 5 to 75 µM with 3–4 replicates each were tested. Therefore, 400 mL of the respective medium was filled into 1 L flasks, autoclaved, and inoculated with *M. aeruginosa* (1.5 × 10^5^ cells mL^−1^), which is similar to the inocula used by Long [62,76]. The experiment lasted for 28 days, and the flasks were randomized daily to compensate for potential heterogeneities in the light regime. Every 1–2 days, samples (0.5 mL) were taken and if necessary, diluted for cell counts using a Neubauer improved counting chamber. Per sample, at least 100–150 cells (at low densities) or 3 large squares (at high densities) were counted to ensure appropriate accuracy. In intervals of 2–4 days, 10 and 200 mL were taken to measure the particulate organic carbon (POC), nitrogen (PON), and phosphorus (P_part_) as well as the PIs. The volume needed for the analyses was roughly estimated based on the cell density.

### 4.2. Determination of POC, PON, and P_part_

POC and PON were measured by filtering a sample volume equivalent to approx. 0.25 mg POC on pre-combusted GF/F filters (Macherey & Nagel, Düren, Germany), which were subsequently dried at 60 °C for at least 24 h. The filters were packed into tin capsules and analyzed using a Flash EA2000 Analyzer (Thermo Fisher Scientific, Waltham, MA, USA). For the analysis of P_part_, 0.5 mg C was filtered on GF/F filters, transferred into 10 mL of a potassium peroxodisulphate and sodium hydroxide, and autoclaved for 1 h at 120 °C; soluble reactive P was subsequently analyzed with the molybdate-ascorbic acid method [77] with a DR5000 UV-Vis spectrometer (Hach, Loveland, CO, USA). The obtained values were used to calculate the molar stoichiometric ratios (C:N, C:P and N:P) of the cyanobacterial biomass.

### 4.3. Extraction and Quantification of PIs

PIs were extracted and measured according to Burberg et al. [24]. Briefly, samples (approx. 0.25 mg C) were centrifuged (5 min). The supernatant was discarded and 10 mL methanol (80%) as well as 10 µL of the internal standard (microcystin LR, 10 µg mL^−1^, Enzo Life Sciences, Farmingdale, NY, USA) were added to the cell pellet. The samples were re-suspended, sonicated, and again centrifuged (3 min, 4500× *g*). The supernatant was evaporated to dryness, re-dissolved in 1 mL methanol, and again dried and taken up in methanol (100 µL). Finally, the samples were centrifuged (2 min, 20,000× *g*), and the supernatant was transferred into HPLC vials.

PIs were quantified using an ultra high pressure liquid chromatography system (UHPLC, Accela, ThermoFisher Scientific, Waltham, MA, USA), coupled with an Exactive Orbitrap mass spectrometer (MS, ThermoFisher Scientific, Waltham, MA, USA). The chromatographic separations were carried out on a C_18_-column (Nucleosil, 125/2, 100-3, Macherey and Nagel, Düren, NRW, Germany) as stationary phase with acetonitrile (A) and ultra-pure water (B), each containing 0.05% trifluoracetic acid (TFA) as mobile phase with the following gradient: 0 min: 20% A, 14 min: 100% A, 16 min: 100% A, 16.5 min: 20% A, 18 min: 20% A. The column temperature was set to 30 °C, the flow rate was 300 µL/min, and the injection volume of the sample was 10 µL.

The MS was operated according to Burberg et al. [24]. Under the applied conditions, the two PIs form two positively charged adduct ions ([M+H-H_2_O]^+^; [M+Na]^+^) with *m*/*z* = 903.46108 and 943.45331 (BN920 adducts) and *m*/*z* = 937.42211 and 977.41394 (CP954 adducts). For further calculations and analyses, the single adducts of each PI were summed up to ´BN920´ and ´CP954´. MC-LR ([M+H]^+^) was measured at *m*/*z* = 995.55604. Peak intensities were extracted from the chromatograms using the R package ´enviMass´ [78] and converted to concentrations via previous established calibration curves. Subsequently, the PI concentrations in the samples were normalized to the culture volume or to the extracted POC, as proxy for the cyanobacterial biomass.

### 4.4. Modeling and Statistical Analyses

All statistical analyses were performed using R [79] and RStudio [80]. Our expectation was that *Microcystis* shows logistic growth. A logistic (sigmoidal) growth curve starts with an initial exponential growth phase, followed by a mid-exponential, and then a stationary phase when one or more resources, e.g., P, become limiting. The R package ‘growthrates’ [81] was used to fit logistic growth models based on POC and *Microcystis* cell abundance. The model equation was:

(1) $$N_{t}=\left(K\cdot N_{0}\right)/{(N}_{0}+\left({K-N}_{0}\right)e^{{-µ}_{\max}t}),$$

with N_0_ as the initial biomass or cell abundance, N_t_ as the biomass or cell abundance at time (t), and the two model parameters carrying capacity (K) and maximal growth rate (µ_max_). Effects of the initial PO_4_^3−^-concentration on K and µ_max_ were tested using one-way ANOVAs followed by HSD post-hoc tests (TukeyHSD). Residuals were tested for normal distribution (Shapiro–Wilk test) and variance homogeneity (Levene test). Growth rates were taken from the logistic growth model for POC, and are therefore based on changes in POC. Thus, they may be referred to as C net production rate. The growth rates were correlated to the PI content which allowed us to analyze how the inhibitor content was affected by growth rate. The effects of sampling day, initial PO_4_^3^^−^-concentration, and sampling day x PO_4_^3−^-concentration on P_part_, stoichiometry and PI content were tested applying linear mixed effects models using the R package lme4 [82].

Linear mixed-effects models were also used to analyze the relationship between PIs and growth rate as well as between PIs and elemental molar ratios. Natural logarithm (ln) respectively square root transformation was used for stabilization of variance and normality of residuals so that the transformed linear regressions can be written as: ln y = a + b $\sqrt{x}$ for the growth rate and ln y = a + b ln x for elemental ratios. To account for a potential nonlinear relationship, a quadratic term was employed [82]:

(2) $$\mathrm{lny}=a+\mathrm{blnx}+c\;{\left(\ln\;x\right)}^{2},$$

in case of a log transformed independent variable (x), and

(3) $$\mathrm{lny}=a+b\sqrt{x}+c\;{\left(\sqrt{x}\right)}^{2}=a+b\sqrt{x}+\mathrm{cx},$$

for square root transformed variables. To account for pseudo-replication within trials, combinations between treatment and replicates were treated as random effect (individual slopes for all 28 combinations of treatment and replicate) while intercept (a), slope (b), and quadratic term (c) describe dependency on the fixed-effect variable ln x respectively $\sqrt{x}$. Models with increasing complexity were fitted, a null model with only the random effects, a linear model, and a quadratic model (Table 5). AIC (Akaike information criterion) values are given as an indicator of model adequacy. *p*-values were estimated by likelihood ratio tests (Chi^2^) between consecutive models (i.e., between null model and linear model resp. between quadratic and linear model). The coefficient of determination (R^2^) accounts for the explanatory contribution of the fixed effect of the models only. In contrast, the Pearson correlation coefficient R_P_ measures direct linear dependency of the transformed data. The effectivity of the transformations was checked graphically: normality of residuals with quantile–quantile plots and variance homogeneity by plotting residuals versus fitted values.

For a comparison of PI content and cellular nutrient quota within growth phases and between P-regimes (high > 30 µM, medium = 15–30 µM, low < 15 µM), data from each combination of growth period and phosphorus treatment were pooled while retaining individual experimental units as replicates. Logistic growth was subdivided into three phases (early exponential, mid-exponential, and late growth) at time points where the modeled POC approached 25% resp. 75% of the carrying capacity (K). The calculation was done for each treatment and replicate separately. Pairwise differences between combinations of growth phase and P-regime were tested post-hoc using generalized linear hypothesis tests [83] of linear models, with P-regime, growth period, and their interaction as explanation variables. A letter-based representation was used to indicate significant and non-significant pairwise comparisons [84] at a significance level of 0.05.

## Figures and Tables

**Table 1:** Modeled growth parameters of *M. aeruginosa* grown with 8 different initial PO_4_^3−^-concentrations. The model was based on cell abundance (Figure 1a). Mean values (±SD, n = 3–4) of the initial cell abundance (N_0_), carrying capacity (K) and maximal growth rate (µ_max_) were determined and tested for differences between treatments (one-way ANOVA followed by a Tukey post-hoc test, *p* = 0.05). Capital letters indicate significant differences between treatments.

PO_4_^3−^ [µM]	N_0_ [10^4^ Cells mL^−1^]	K [10^4^ Cells mL^−1^]	µ_max_ [d^−1^]	R_2_ Fits ^1^
**5**	24.7 ± 3.3	1022 ± 63 ^A^	0.34 ± 0.03 ^AB^	0.82
**10**	14.8 ± 4.5	1272 ± 172 ^A^	0.33 ± 0.02 ^AB^	0.87
**15**	18.2 ± 18.7	4718 ± 362 ^C^	0.45 ± 0.09 ^A^	0.96
**20**	7.28 ± 7.24	4284 ± 247 ^BC^	0.44 ± 0.06 ^A^	0.97
**30**	51.7 ± 14.8	4328 ± 381 ^BC^	0.27 ± 0.04 ^B^	0.94
**40**	62.9 ± 32.6	3729 ± 516 ^B^	0.25 ± 0.04 ^B^	0.92
**50**	7.9 ± 6.5	4129 ± 484 ^BC^	0.43 ± 0.1 ^A^	0.93
**75**	23.9 ± 19.9	4312 ± 239 ^BC^	0.33 ± 0.06 ^AB^	0.94

^1^ General fit equation: $N_{t}=\left(K\cdot N_{0}\right)/(N_{0}+({K-N}_{0})\;e^{{-µ}_{\max}t})$ with t = time [d], N_t_ = cell abundance at time t [cells mL^−1^].

**Table 2:** Modeled growth parameters of *M. aeruginosa* grown with 8 different initial PO_4_^3−^-concentrations. The model was based on POC (Figure 1b). Mean values (± SD, n = 3–4) of the initial POC concentration (N_0_), carrying capacity (K), and maximal growth rate (µ_max_) were determined and tested for differences between treatments (one-way ANOVA followed by a Tukey post-hoc test, *p* = 0.05). Capital letters indicate significant differences between treatments.

PO_4_^3^ [µM]	N_0_ [µg C mL^−1^]	K [µg C mL^−1^]	µ_max_ [d^−1^]	R^2^ Fits ^1^
**5**	5.02 ± 1.17	93.1 ± 3.6 ^A^	0.24 ± 0.03 ^A^	0.98
**10**	1.17 ± 0.13	106.2 ± 4.6 ^AB^	0.32 ± 0.004 ^AB^	0.98
**15**	2.92 ± 1.62	272.9 ± 9.2 ^CD^	0.41 ± 0.04 ^BCD^	0.99
**20**	0.32 ± 0.18	230.8 ± 5.5 ^BC^	0.5 ± 0.03 ^D^	0.99
**30**	1.07 ± 0.84	345.1 ± 20.4 ^E^	0.49 ± 0.05 ^D^	0.92
**40**	3.82 ± 2.33	415 ± 67.3 ^F^	0.37 ± 0.07 ^BC^	0.97
**50**	0.03 ± 0.008	327.5 ± 6.8 ^DE^	0.67 ± 0.02 ^E^	0.99
**75**	0.5 ± 0.28	473.4 ± 9.6 ^F^	0.45 ± 0.04 ^CD^	0.99

^1^ General fit equation: $N_{t}=\left(K\cdot N_{0}\right)/(N_{0}+({K-N}_{0})\;e^{{-µ}_{\max}t})$ with t = time [d], N_t_ = POC at time t [µg C mL^−1^].

**Table 3:** Linear mixed-effects model for the particulate phosphorus (P_part_; Figure 1c) and biomass stoichiometry (C:N, C:P, N:P; Figure 2) of *M. aeruginosa* during a 28 days growth experiment. Mean Sq: mean square, NumDF: numerator degrees of freedom, DenDF: denominator DF, F value: test statistic.

	Mean Sq	NumDF	DenDF	F Value	*p*-Value ^1^	
**P_part_**						
**Treatment**	1.18 × 10^−7^	7	261.25	0.6286	0.7321	
**Day**	5.27 × 10^−5^	1	260.22	280.27	<2 × 10^−16^	***
**Treatment x Day**	3.53 × 10^−6^	7	260.22	18.74	<2 × 10^−16^	***
**C:N-ratio**						
**Treatment**	138.742	7	197.87	67.057	<2.2 × 10^−16^	***
**Day**	250.839	9	197.07	121.265	<2.2 × 10^−16^	***
**Treatment x Day**	26.879	63	197.07	12.994	<2.2 × 10^−16^	***
**C:P-ratio**						
**Treatment**	614,724	7	197.02	54.6476	<2.2 × 10^−16^	***
**Day**	1,364,784	9	197.22	121.3772	<2.2 × 10^−16^	***
**Treatment x Day**	32,240	63	197.22	2.8673	1.323 × 10^−08^	***
**N:P-ratio**						
**Treatment**	12,892.4	7	197.97	110.690	<2.2 × 10^−16^	***
**Day**	8209.8	9	197.06	70.502	<2.2 × 10^−16^	***
**Treatment x Day**	724.3	63	197.06	6.220	<2.2 × 10^−16^	***

^1^ Significance levels: *** <0.001.

**Table 4:** Linear mixed-effects model for effects of treatment and sampling day on the protease inhibitor content of BN920 and CP954 in *M. aeruginosa*. Mean Sq: mean square, NumDF: numerator degrees of freedom, DenDF: denominator DF, F value: test statistic.

	Mean Sq	NumDF	DenDF	F Value	*p*-Value ^1^	
**BN920**						
**Treatment**	71.715	7	197.36	18.2154	<2.2 × 10^−16^	***
**Day**	172.494	9	197.14	43.8287	<2.2 × 10^−16^	***
**Treatment x Day**	14.333	63	197.14	3.6419	2.744 × 10^−12^	***
**CP954**						
**Treatment**	4408.5	7	197.47	53.8364	<2.2 × 10^−16^	***
**Day**	2508.0	9	197.12	30.6383	<2.2 × 10^−16^	***
**Treatment x Day**	342.5	63	197.12	4.1837	8.755 × 10^−15^	***

^1^ Significance levels: *** <0.001.

**Table 5:** Equations, parameters, and significance tests for the regression models applied to the data in Figure 4 and Figure 5. Data were natural logarithmic (ln) resp. square root transformed and then analyzed using linear mixed-effects models with a single explanation variable (x) as fixed effect (intercept, slope and quadratic term) and individual slopes for the 28 treatment x replicate combinations. The null model (-) contains the same random effects but omitted the explanation variable. *p*-values indicate significant likelihood ratio (Chi^2^) of consecutive models (i.e., between linear and null model resp. between quadratic and linear model). R_p_: Pearson correlation coefficient, calculated from the transformed x and y data: R^2^: coefficient of determination of the mixed model (fixed effects only). AIC: Akaike information criterion.

	Euation	R_p_	R^2^	AIC	*p*-Value ^1^	
**BN920**						
**-**	ln y = 1.576			497.1		-
**µmax**	ln y = 1.072 + 0.9555 $\sqrt{x}$	0.37	0.13	493.1	1.00 × 10^−6^	***
**µmax**	ln y = 0.9178 + 2.578 $\sqrt{x}$ − 2.28 x		0.14	485.3	3.6 × 10^−4^	**
**C:N ratio**	ln y = 3.098 − 0.7396 ln x	−0.43	0.18	442.3	1.20 × 10^−14^	***
**C:N ratio**	ln y = 6.426 − 3.612 ln x + 0.6036 (ln x)^2^		0.21	439.7	0.018	*
**C:P ratio**	ln y = 2.916 − 0.2622 ln x	−0.38	0.14	449.0	2.00 × 10^−13^	***
**C:P ratio**	ln y = 3.179 − 0.3684 ln x + 0.01027 (ln x)^2^		0.14	456.3	0.7	
**N:P ratio**	ln y = 2.301 − 0.2586 ln x	−0.24	0.05	473.6	4.60 × 10^−8^	***
**N:P ratio**	ln y = 2.838 − 0.6473 ln x + 0.06414 (ln x)^2^		0.09	477.5	0.081	
**CP954**						
**-**	ln y = 2.768			549.1		-
**µmax**	ln y = 2.989 − 0.03925 $\sqrt{x}$	−0.07	0.002	597.5	0.83	
**µmax**	ln y = 2.591 + 4.081 $\sqrt{x}$ − 5.75 x		0.11	557.9	2.40 × 10^−10^	***
**C:N ratio**	ln y = 2.602 + 0.1626 ln x	−0.009	−0.01	565.0	0.16	
**C:N ratio**	ln y = −7.357 + 8.758 ln x − 1.807 (ln x)^2^		0.10	533.8	5.60 × 10^−9^	***
**C:P ratio**	ln y = 1.736 + 0.2134 ln x	0.37	0.13	533.5	1.50 × 10^−7^	***
**C:P ratio**	ln y = −1.478 + 1.515 ln x − 0.126 (ln x)^2^		0.14	524.6	6.10 × 10^−5^	***
**N:P ratio**	ln y = 1.896 + 0.3076 ln x	0.42	0.18	519.5	1.70 × 10^−9^	***
**N:P ratio**	ln y = 1.135 + 0.8565 ln x − 0.09022 (ln x)^2^		0.17	521.1	0.026	*

^1^ Significance levels: *** <0.001, ** <0.01, * <0.05.
